# Transmission Characteristics of Barley Yellow Striate Mosaic Virus in Its Planthopper Vector *Laodelphax striatellus*

**DOI:** 10.3389/fmicb.2018.01419

**Published:** 2018-06-29

**Authors:** Qing Cao, Wen-Ya Xu, Qiang Gao, Zhi-Hao Jiang, Song-Yu Liu, Xiao-Dong Fang, Dong-Min Gao, Ying Wang, Xian-Bing Wang

**Affiliations:** ^1^State Key Laboratory of Agrobiotechnology, College of Biological Sciences, China Agricultural University, Beijing, China; ^2^College of Plant Protection, China Agricultural University, Beijing, China

**Keywords:** *Barley yellow striate mosaic virus*, cytorhabdovirus, persistent-propagative virus, *Laodelphax striatellus*, hindgut

## Abstract

The most economically important plant viruses are specifically transmitted by phytophagous insects that significantly affect viral epidemiology. *Barley yellow striate mosaic virus* (BYSMV), a member of the genus *Cytorhabdovirus*, is transmitted by the small brown planthopper (SBPH, *Laodelphax striatellus*) in a persistent-propagative manner. However, the infection route of BYSMV in SBPHs is poorly understood. In this study, immunofluorescence confocal laser scanning microscopy (iCLSM) was performed to investigate the route of BYSMV in SBPHs. We unexpectedly found that BYSMV initially infected the hindgut epithelium of SBPHs, instead of the midgut epithelium initially infected by other persistent-propagative viruses. Subsequently, BYSMV disseminated to the hindgut visceral muscles and spread to other parts of alimentary canals, hemolymph, and salivary glands. Comparative analysis of gene expression on viral mRNAs and the BYSMV nucleoprotein by using different molecular detection and immunohistochemistry further demonstrated that BYSMV initially infected and replicated in the hindgut epithelial cells of SBPHs. Collectively, our study provides the first insight into that hindgut is initial infection site of BYSMV that represents a new dissemination route of persistent-propagative viruses.

## Introduction

To survive in sessile plant hosts, most plant-infecting viruses rely on specific vectors for plant-to-plant transmission in nature. Although some fungi, nematodes, and various types of invertebrates serve as vectors for some plant viruses, the majority of described plant viruses are transmitted by sap-feeding hemipteran insects including aphids, leafhoppers, planthoppers, and whiteflies (Hogenhout et al., 2008). Based on acquisition and inoculation thresholds, and retention of viruses by insect vectors, transmission relationships between insect vectors and plant viruses are grouped into four types: non-persistent, semi-persistent, persistent-circulative, and persistent-propagative (Ng and Falk, 2006; Hogenhout et al., 2008; Blanc et al., 2014). The members of rhabdoviruses, orthotospoviruses, tenuiviruses, and reoviruses are transmitted by specific insect vectors in a persistent-propagative manner (Hogenhout et al., 2008). Persistent-propagative viruses usually multiply in epithelial cells of the alimentary canal, and then pass through the gut wall to hemolymph, and finally move to salivary glands from where the viruses are introduced into healthy susceptible plant hosts (Ammar et al., 2008; Hogenhout et al., 2008). In addition, some persistent-propagative viruses, including rhabdoviruses, reoviruses, and tenuiviruses are transmitted vertically from parents to their progenies (Nault and Ammar, 1989; Falk and Tsai, 1998; Ammar et al., 2008; Wei and Li, 2016). Persistent-propagative viruses replicate and spread in their insect vectors requiring specific interaction of viral components and vector factors to overcome several major transmission barriers including midgut infection and escape barriers, salivary gland infection and escape barriers, and transovarial transmission barriers (Ammar et al., 2008; Hogenhout et al., 2008).

In competent planthopper vectors, persistent-propagative viruses must first efficiently infect the midgut epithelial cells, escape the midgut, spread to hemolymph, and finally breach the basal laminae of the salivary gland into salivary secretions that facilitate the injection of the virus into a susceptible host during insect feeding. The failure of virus transmission in incompetent insect vectors may be due to the impede of viral entrance, replication, or dissemination in insect cells or organs (Ammar, 1994; Jackson et al., 2005; Ammar et al., 2008; Whitfield et al., 2015). Midgut barriers are the first barriers and principle determinants of vector competence. Many persistent viruses are restricted to midgut epitheliums of incompetent insect vectors probably due to lack of viral receptors or some specific immune response to the viruses (de Assis et al., 2005; Ding, 2010; Donald et al., 2012; Jia et al., 2012a; Chen et al., 2015; Neelakanta and Sultana, 2016; Whitt et al., 2016). Silencing of the core component Dicer-2 of the siRNA pathway allows the efficient propagation within the midgut epithelium and transmission of *Southern rice black streaked dwarf virus* (SRBSDV) by its incompetent vector (Lan et al., 2015).

Plant rhabdoviruses initially fall into two genera, *Nucleorhabdovirus* and *Cytorhabdovirus*, which mature in the nucleus and cytoplasm of host cells, respectively. Besides, the *Dichorhavirus* and *Varicosavirus* genera are recently recognized as rhabdoviruses based on significant genome sequence identities with plant rhabdoviruses, even though they contain bipartite negative-sense ssRNA genomes (Dietzgen et al., 2017). Plant rhabdoviruses are usually transmitted by hemipteran insects, including aphids, leafhoppers, or delphacid planthoppers in a persistent-propagative manner (Jackson et al., 2005; Ammar et al., 2008). Previous immunofluorescence microscopy studies have shown that *Maize mosaic virus* (MMV), a member of *Nucleorhabdovirus* genus, initially infects midgut and anterior diverticulum, and then spread to other tissues including nervous system in *Peregrinus maidis* (Ammar el and Hogenhout, 2008). The understanding of virus-vector interactions in plant cytorhabdoviruses is largely unknown.

*Barley yellow striate mosaic virus* (BYSMV), a member of *Cytorhabdovirus* genus, is transmitted by the small brown planthopper, *Laodelphax striatellus* (*L. striatellus*), in a persistent-propagative manner (Conti and Plumb, 1977; Conti, 1980). BYSMV was first isolated from planthoppers in Italy (Conti, 1969), and subsequently reported in other countries (Bassi et al., 1980; Izadpanah et al., 1991; Makkouk et al., 2004; Almasi et al., 2010; Di et al., 2014; Yan et al., 2015). Recently, we isolated BYSMV from the wheat fields in northern China (Di et al., 2014) and obtained the complete genome (Yan et al., 2015). The BYSMV genome consists of 12,706 nucleotides and encodes ten proteins in the order 3′-N (nucleoprotein)-P (phosphprotein)-P3-P4/P5-P6-M (matrix protein)-G (glycoprotein)-P9-L (polymerase)-5′ (Yan et al., 2015). The N protein mainly functions to encapsidate the genomic/antigenomic RNA acting templates of virus replication and transcription (Jackson et al., 2005). Thus, the mRNAs and proteins accumulation of the rhabdovirus N proteins are usually detected and analyzed to represent virus infection.

In the present work, we used immunofluorescence staining and molecular analysis to investigate the temporal and spatial distribution of BYSMV within *L. striatellus*. We found that the hindguts of *L. striatellus* vectors rather than their midguts were acquisition sites for BYSMV and provided molecular evidence that BYSMV could replicate in the cytoplasm of hindgut epithelia of *L. striatellus*.

## Materials and Methods

### Insect Rearing and Virus Maintenance

Planthopper vectors, *L. striatellus*, were isolated from Hebei province, China and maintained in growth chambers for nearly 4 years. The Chinese isolate of BYSMV was maintained on diseased wheat plants through serial transmission by *L. striatellus* as described previously (Di et al., 2014; Yan et al., 2015). Both viruliferous and healthy *L. striatellus* were reared separately on wheat seedlings in growth chambers with a 16 h (h) light/8 h dark and held at 25 ± 2°C during light and 20 ± 2°C during dark periods.

### Polyclonal Antibody Preparation

The BYSMV N (GenBank: NC_028244.1) and actin genes (GenBank: KC683802.1) were amplified using specific primers of BYSMV N (5′ GGAATTCCATATGATGGAAGAAGATCATGG 3′ and 5′ CCGCTCGAGGGAGAAGATCTGGTCAGCATT 3′) and Actin (5′ GGAATTCAACATCTGCTGGAAGGTGGAGAGG 3′ and 5′ CATGCCATGGCTCTGTACGCCTCCGGTCGTAC 3′), and then engineered into pET-30a (+) vector. The resulting plasmids pET-30a-N and pET-30a-Actin were transformed into the Rosetta strain of *Escherichia coli.* BYSMV N and Actin proteins were purified from the final suspension of transformed cell treated using Ni-NTA resin (Qiagen, Hilden, Germany) as previous report (Dong et al., 2016). The purified proteins immunized rabbits, and the specific polyclonal antisera was used to purify Immunoglobulin G (IgG) using A-Sepharose affinity column (Sigma–Aldrich).

### The Acquisition Efficiency of BYSMV by *L. striatellus*

Second-instar nymphs of *L. striatellus* (*n* = 50, three biological repetitions) were allowed 1, 4, 24, 36, and 48 h acquisition access period (AAP) on BYSMV infected wheat plants. Insects were incubated on healthy rice seedings for a 12-day inoculation period (IP), and then examined by iCLSM (immunofluorescence confocal laser scanning microscopoy). The planthoppers fed on healthy wheat plants were acted as negative controls.

### Immunofluorescence Confocal Laser Scanning Microscopy (iCLSM)

Second-instar nymphs of planthoppers were allowed a 36-h AAP on diseased wheat plants infected with BYSMV. After virion acquisition, planthoppers were transferred to healthy rice seedlings, and changed fresh seedings every 7 days to assure sufficient nutrition. At different days after the AAP, alimentary canal of planthoppers were dissected, fixed in 4% paraformaldehyde overnight at 4°C, and washed in 0.01 M PBS buffer (pH 7.4). Then, the organs were permeabilized in PBS buffer harboring 2% Triton X-100 at 30°C for 30 min. After washed in PBS buffer, the organs were stained with BYSMV N protein antibody conjugated directly to fluorescein-5-isothiocyanate (FITC, Sigma–Aldrich) for 2 h at 37°C. To distinguish muscle fibers from other tissues, actin was stained with phalloidin–rhodamine (Invitrogen). Finally, the stained products were washed with PBS buffer and processed for Olympus immunofluorescence microscopy (Olympus FV1000). The organs dissected from heathy planthoppers were stained as negative controls. DAPI, GFP, and RFP fluorescence were visualized under 405, 488, and 543 nm, respectively. The value of gain and offset is set to 1%. The high voltage is set to 400–600. To avoid cross fluorescence effects, a line sequential scanning mode was used for image capture at a 1024 × 1024 pixel resolution.

### Hemolymph Collection and Immunofluorescence Staining

Hemolymph collection and immunofluorescence staining were performed by the methods as reported previously (Wang et al., 2014; Huo et al., 2018). Briefly, SBPHs were anesthetized on ice for 30 min. The forelegs at the coxa-trochanter joint were severed by forceps. The hemolymph was disembogued and drawn onto the tip of forceps. The collected hemolymph was quickly washed in PBS drops, where the drops were on poly (L-lysine)-coated cover glasses. The glasses were incubated at 37°C for 30 min to allow the adhesion of hemocytes. The hemolymph was fixed in 4% paraformaldehyde for 30 min at room temperature and washed in 0.01 M PBS buffer (pH 7.4). Subsequently, hemolymph was permeabilized in PBS buffer harboring 0.2% Triton X-100 for 5 min at room temperature and stained with BYSMV N-FITC antibody (1:100). Finally, stained products were washed with PBS buffer and processed for Olympus immunofluorescence microscopy (Olympus FV1000). The planthoppers fed on healthy wheat plants were used as negative controls.

### Western Blotting

At 4 and 8 days after AAP, the alimentary canals of 50 planthoppers were dissected and separated into two parts, hindgut tissues and the remaining tissues (including esophagus, anterior diverticulum, midgut, and malpighian tubules). Total proteins were isolated from the two parts of alimentary canals in SDS buffer [100 mM Tris-HCl (pH 6.8), 20% glycerol, 4% SDS, 0.2% bromophenol blue, 10% β-mercaptoethanol] for western blotting, separated in SDS-PAGE gels, and transferred onto nitrocellulose membranes. Anti-N (1:3000) polyclonal antibody was used to quantify the accumulations of the BYSMV N proteins, as described previously (Yan et al., 2015). Anti-actin (1:1000) polyclonal antibody was used to quantify actin accumulations as internal loading control.

### RNA Analysis

Total RNA was isolated from the hindgut tissues and the remaining tissues of the alimentary canals of 200 planthoppers. The RNA was quantified using a NanoDrop ND-1000 (Thermo Fisher Scientific). Total RNA (1.5 μg) was reverse-transcribed by using M-MLV Reverse Transcriptase (Promega) with oligo (dT) 20 primer and the produced cDNAs were used as templates for semi-quantitative RT-PCR and RT-qPCR. Expression levels of N, P, and M genes of BYSMV were examined by semi- quantitative RT-PCR with primers of N (5′GAAGATCATGGATTGGACAGAGAG-3′; 5′GCAGGAGTGTAAACCGGGAT3′), P (5′AGATGGGATCTTCGGTGAGC-3′; 5′CTTCCACACCGGAGATATACC3′), M (5′GGAGGTAGACTTTGGAGAAGGAG 3′; 5′CGAAGATCCAGAGTAAGAGCT3′) according to the manufacturers’ instructions. The actin gene of *L. striatellus* served as an internal control with the specific primers (5′GCCCATCTACGAAGGTTAC3′; 5′CCATTTCCTGTTCGAA GTCCAG3′). The semi-quantitative RT-PCR was performed using the following thermal cycling profile: 94°C for 10 min, followed by 25 cycles of amplification (94°C for 30 s, 55°C for 30 s, and 72°C for 30 s), and 72°C for 10 min. The relative intensity of each lane was quantified using Quantity One software. The RT-qPCR assay was performed on a BIO-RAD CFX96^TM^ Real-Time System (Bio-Rad) using the 1 X SsoFastTM EvaGreen Supermix (Bio-Rad), according to the manufacturers’ instructions. The cycling parameters were: 95°C for 3 min, followed by 48 cycles of 95°C for 10 s and 55°C for 20 s. Actin gene of *L. striatellus* was used as an internal control.

### Immunohistochemistry

Hindgut samples were dehydrated in ethanol series and embedded in paraffin wax. Serial sections (8 μm) were mounted on slides coated with poly-L-lysine, incubated with 10% normal donkey serum to reduce background, and incubated with the primary antibody (1:200) raised against BYSMV N protein at 4°C for 12 h. After washed three times in PBS buffer, resulting sections were incubated with a secondary antibody (1:50), donkey anti-rabbit lgG conjugated with Alexa fluor^®^ 488 at 25°C for 40 min, and then processed for Olympus immunofluorescence microscopy (Olympus FV1000). Hindguts of healthy *L. striatellus* were used as negative controls.

## Results

### BYSMV Acquisition Ability of *L. striatellus*

**FIGURE 1:**
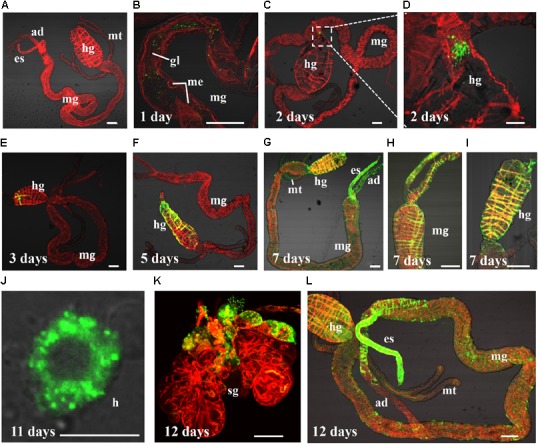
*Barley yellow striate mosaic virus* (BYSMV) infection starts in the hindgut of SBPHs. Internal organs of infected SBPHs were immunolabeled for BYSMV with N-FITC (green) and stained for actin with phalloidin-rhodamine (red), then examined by confocal microscopy. **(A)** View of dissected alimentary canal of a negative control planthopper unexposed to BYSMV. At 1 **(B)**, 2 **(C,D)**, 3 **(E)**, 5 **(F)**, 7 **(G–I)**, 11 **(J)**, and 12 **(K,L)** days padp, internal organs of BYSMV-infected SBPHs were dissected, isolated, and processed for iCLSM. These images are representative of multiple experiments with multiple preparations. ad, anterior diverticulum; mg, midgut; hg, hindgut; mt, malpighian tubules; es, esophagus; sg, salivary gland; h, hemocytes; gl, gut lumen; me, midgut epithelium. Bar in hemocytes is equal to 10 μm. Other Bars, 100 μm.

To determine acquisition efficiency of BYSMV by its planthopper vector, we allowed the second-instar nymphs of non-viruliferous SBPH populations to feed on BYSMV-infected wheat plants for 1, 4, 24, 36, and 48 h AAP. These insects were reared on healthy wheat seedlings for a 12-day incubation period. Internal organs of about 50 insects were dissected and stained by BYSMV N protein-specific IgG directly conjugated with fluorescein isothiocyanate (N-FITC) for iCLSM. As negative controls, the internal tissues of SBPHs exposed to healthy plants did not show specific fluorescence (**Figure 1A** and **Supplementary Figure S1**). In contrast, the BYSMV-infected alimentary canals showed high intensity of GFP signal that represented accumulation of the BYSMV N protein (**Supplementary Figure S1**).

Results from three independent repetitions with a total number of 134 –151 insects showed that 2.7%, 9.7%, 25.2%, 45.9%, and 49.3% of SBPHs were infected by BYSMV with 1, 4, 24, 36, and 48 h AAPs, respectively (**Table 1**). The results indicated that *L. striatellus* could acquire BYSMV after a feeding period of 1 h, and the BYSMV acquisition rates of nymphs increased to 49.3% after 48 h feeding (**Table 1**).

**Table 1:** Acquisition access period (AAP) and BYSMV-positive rate of SPBHs.

AAP	Infectivity (No. of infected/total insects)			
	Experiment 1	Experiment 2	Experiment 3	Total (percentage)
1 h	1/49	1/50	2/50	4/149(2.7%)
4 h	5/50	6/50	3/45	14/145(9.7%)
24 h	12/50	13/50	13/51	38/151(25.2%)
36 h	30/53	18/43	19/50	67/146(45.9%)
48 h	27/50	20/43	19/41	66/134(49.3%)

*Second-instar nymphs of SBPHs were fed on BYSMV-infected barley plants for different time acquisition access, then incubated on healthy barley seedling for 12 days. The whole alimentary canals were dissected for iCLSM test using BYSMV-N antibody conjugated with fluorescein isothiocyanate (N-FITC)*.

### BYSMV Infection Starts in the Hindgut of *L. striatellus*

To determine the temporal and spatial distribution of BYSMV in its vector, we allowed second-instar nymphs of SBPH to feed on diseased barley plants for a 36-h AAP. Then, the whole internal organs of 50 planthoppers were dissected and stained by N-FITC and the actin dye phalloidin–rhodamine (Invitrogen) for iCLSM.

Results of three independent repetitions (*n* = 50) are summarized in **Table 2**. The micrographs illustrating key events in BYSMV dissemination route in SPBH were shown in **Figure 1**. At 1-day post-first access to diseased plants (padp), ingested BYSMV were labeled as punctate structures throughout the gut lumen (**Figure 1B**). One day later, a sign of specific labeling of BYSMV antigen was only observed in the hindgut epithelium in about 37.8% of SBPHs examined (**Figures 1C,D** and **Table 2**). At this time, no detectable virus accumulation was found in any other tissues of alimentary canal (**Figure 1C**). At 3-day padp, BYSMV N was detected in a high proportion of insect hindgut tissues (up to 42%), and some signals had disseminated to the hindgut visceral muscles from the initial infection sites (**Figure 1E** and **Table 2**). At 5-day padp, BYSMV subsequently spread to the longitudinal and circular muscle cells surrounding hindgut tissues in about 52% of SBPHs examined (**Figure 1F** and **Table 2**). The individual fluorescence panels of 1, 2, 3, and 5 padp were shown in **Supplementary Figure S2**. Simultaneously, a relative low ratio of midgut (up to 22%), esophagus (up to 8.7%), and anterior diverticulum (up to 4.3%) tissues had been infected by BYSMV (**Table 2**).

**Table 2:** Statistics of BYSMV antigens in various organs/tissues of SBPHs.

Days padp	No. positive insects with N-FITC in different tissues					
	Hindgut	Midgut	Esophagus	Anterior diverticulum	Salivary glands	Total number
2 days	17	0	0	0	0	45
3 days	21	2	0	0	0	50
5 days	24	10	4	2	0	46
7 days	24	23	17	10	7	50
12 days	26	26	26	26	25	47

*Second-instar nymphs of SBPHs were fed on BYSMV-infected barley plants for a 36-h acquisition access, then incubated on healthy barley seedling for 2, 3, 5, 7, and 12-day padp. The whole internal organs were dissected for iCLSM test using BYSMV-N antibody conjugated with fluorescein isothiocyanate (N-FITC)*.

In the following observation, BYSMV N was extensively detected in the alimentary canals of insects, including esophagus, anterior diverticulum, midgut, malpighian tubule, and hindgut at 7-day padp (**Figures 1G–I**). At 12-day padp, BYSMV was detected in the hemocytes, salivary glands, as well as in other tissues of about 50% insects examined (**Figures 1J–L** and **Table 2**). The individual fluorescence panels of 7, 11, and 12 padp were shown in **Supplementary Figure S3**. Collectively, BYSMV initially infected the hindgut epithelium of SBPH nymphs, which is distinguishable from other persistent-propagative viruses those establish their initial infection in midgut epithelium of their vectors (Hogenhout et al., 2008; Whitfield et al., 2015; Neelakanta and Sultana, 2016; Wei and Li, 2016). To our knowledge, these observations reveal a new tissue tropism of persistent-propagative viruses in which uptake of viruses occurs in the hindgut of insect vectors.

### Infection of BYSMV in Cytoplasm of SBPH Hindgut Epithelium

**FIGURE 2:**
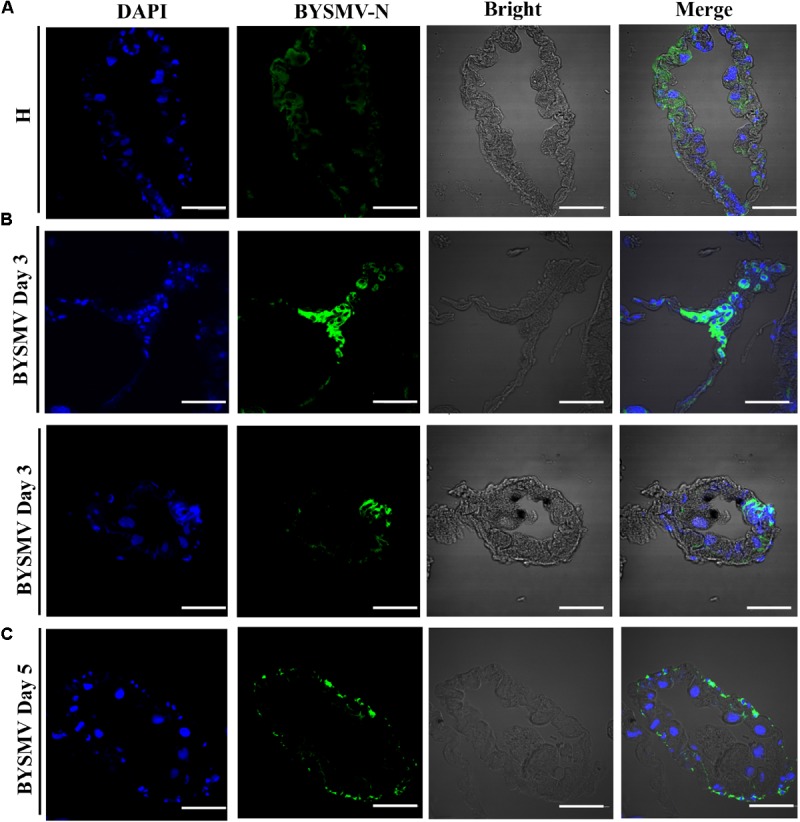
*Barley yellow striate mosaic virus* accumulations in the cytoplasm of the hindguts of viruliferous planthopper vectors. The paraffin sections of the infected hindguts were immunolabeled for BYSMV with N antigens-specific IgG conjugated directly to fluorescein-5-isothiocyanate (green) and stained for nuclei with DAPI (blue). **(A)** The hindgut sections exposed to healthy plants served as negative controls. **(B)** At 3-day padp, BYSMV accumulations in the cytoplasm of epithelial cells of the hindguts of viruliferous planthopper vectors. Upper panel was vertical section; bottom panel was cross section. **(C)** At 5-day padp, BYSMV accumulations in the visceral muscles surrounding the infected hindguts. Bars, 50 μm.

To determine whether the detected BYSMV antigens were from replicating BYSMV or BYSMV-bearing undigested foods, the 6–8 μm thick paraffin sections of viruliferous *L. striatellus* hindguts were processed for immunohistochemistry. Paraffin sections of healthy *L. striatellus* hindguts were used as negative control (**Figure 2A**). At 3-day padp, BYSMV were detected in the cytoplasm of hindgut epithelial cells (**Figure 2B**). At 5-day padp, BYSMV has spread to the visceral muscles surrounding the hindguts (**Figure 2C**), which is consistent with the observation of the whole organs described above (**Figure 1**). These results were further confirmed by using three-dimensional rendered confocal images (**Supplementary Figure S4**). Collectively, these results indicate that the observed fluorescence is due to authentic virus infections but not undigested BYSMV particles.

### The Multiplication of BYSMV in the Hindgut of SBPHs

**FIGURE 3:**
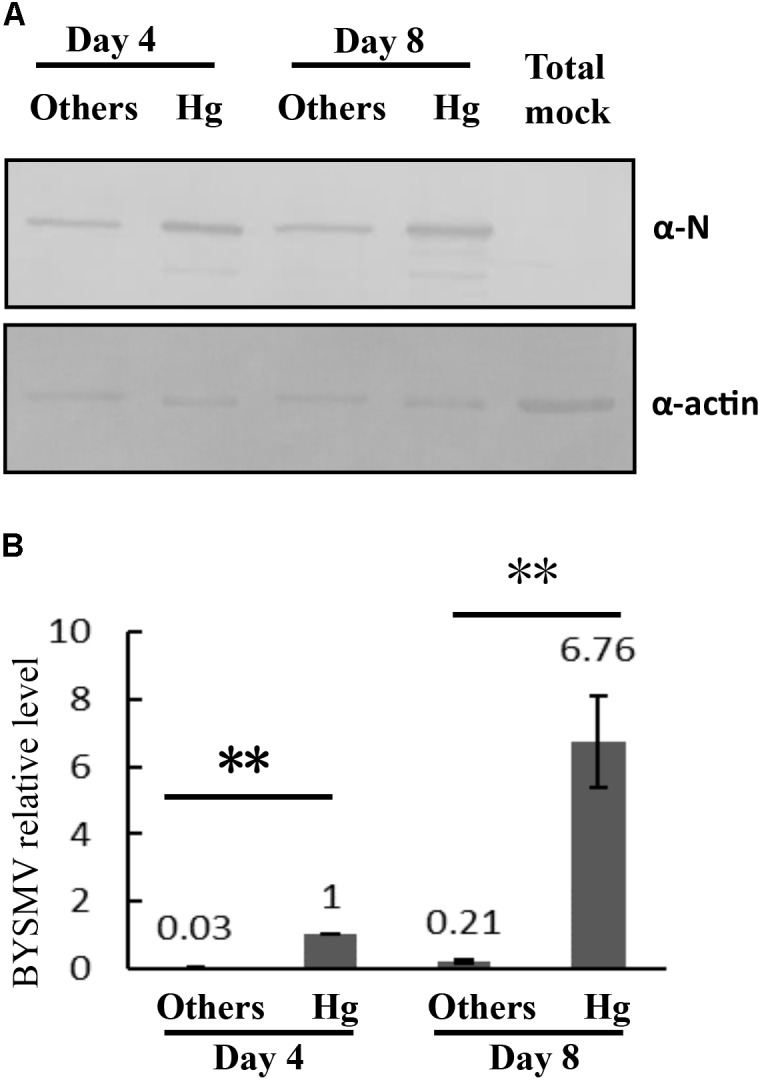
The propagation of BYSMV in *L. striatellus*. **(A)** The virus accumulations of infected hindguts and other tissues of the alimentary canals at 4-day and 8-day padp were verified by western blotting analysis with anti-N polyclonal antiserum. The whole alimentary canals of viruliferous SBPHs served as negative controls (Mock). The actin of *L. striatellus* was detected as the loading controls. **(B)** RT-qPCR assay detecting relative accumulation of BYSMV-N mRNA in the infected hindguts and other tissues of the alimentary canals at 4-day and 8-day padp, respectively. The viral expression level of 4-day-hindgut was normalized as the relative expression level, which was set to 1 data points are the mean value of three independent experiments. The significant difference was analyzed by Student’s *t*-test. ^∗∗^*P*-value < 0.01. Actin was used as an internal control. Other tissues of infected alimentary canals included esophagus, anterior diverticulum, midguts, and malpighian tubules.

To further confirm the propagation of BYSMV in hindgut tissues, the accumulation of BYSMV N protein in hindgut tissues and the other parts of alimentary canals were detected by Western blotting analysis using the BYSMV N specific antibody. At 4-day padp, the N protein was easily detected in hindgut tissues rather than in the other parts of the alimentary canals, indicating that BYSMV established the initial infection in hindgut tissues (**Figure 3A**). At 8-day padp, higher amount of N proteins was accumulated in the hindgut tissues compared with those of at 4-day padp (**Figure 3A**). While, the N protein was detected at a relative low level in the remaining tissues at 8-day padp, indicating BYSMV begin to spread into these tissues at this time (**Figure 3A**).

We further used a quantitative real-time PCR assay to confirm the mRNA level of BYSMV N gene. To rule out DNA contamination, the DNase I treated RNA samples without reverse transcription (Non-RT) were directly used as templates for PCR. The result showed that no DNA bands were amplified from the Non-RT samples (**Supplementary Figure S5A**), indicating the contaminated DNA was treated by DNase I completely. At 4-day and 8-day padp, the accumulation levels of N mRNA in the hindguts were significantly higher than other parts of alimentary canals, and rapidly increased in the hindgut tissues (**Figure 3B**). In semi-quantitative RT-PCR assays, the mRNAs of BYSMV N, P, and M genes were easily detected at 4-day padp, and significantly increasing at 8-day padp in the RNA samples of infected hindgut tissues (**Supplementary Figure S5B**, lane 2 and 4). In contrast, the mRNAs of BYSMV N, P, and M genes in other tissues were under detectable (**Supplementary Figure S5B**, lane 3 and 5). Collectively, these results suggest that BYSMV initially infected and propagated in the *L. striatellus*.

## Discussion

The plant viruses of agricultural importance are usually transmitted by plant-feeding hemipteran insects including aphids, planthoppers, leafhoppers, whiteflies. Persistent-propagative viruses have distinct tissue tropism and infection route in their insect vectors (**Table 3**). For example, rice dwarf virus (RDV) and rice gall dwarf virus (RGDV) are initially infected filter chamber epithelium of their leafhopper vectors (Chen et al., 2011; Zheng et al., 2015). Southern rice black streaked dwarf virus (SRBSDV), rice ragged stunt virus (RRSV), rice stripe virus (RSV), rice grassy stunt virus (RGSV), and tomato spotted wilt virus (TSWV) initially infected midgut epithelial cells of their insect vectors (Kritzman et al., 2002; Jia et al., 2012b,c; Wu et al., 2014; Zheng et al., 2014). Maize mosaic virus (MMV), a member of *Nucleorhabdovirus*, initially infect the midgut and anterior diverticulum of its planthopper vector (Ammar el and Hogenhout, 2008). Here, we unexpectedly discovered that the hindgut of the planthopper vector was the acquisition site for BYSMV. To the best of our knowledge, this is the first time to reveal a new tissue tropism of persistent-propagative viruses in which uptake of viruses occurs in the hindgut of insect vectors.

**Table 3:** Initial infection sites of plant persistent-propagative viruses.

Genus	Virus name	Type of vector	Initial entry	Reference
*Cytorhabdovirus*	*Barley yellow striate mosaic virus* (BYSMV)	Planthopper	Hindgut	–
*Nucleorhabdovirus*	*Maize mosaic virus* (MMV)	Planthopper	Anterior diverticulum, Midgut	Ammar el and Hogenhout, 2008
*Tenuivirus*	*Rice grassy stunt virus* (RGSV)	Planthopper	Midgut	Zheng et al., 2014
*Tenuivirus*	*Rice stripe virus* (RSV)	Planthopper	Midgut	Wu et al., 2014
*Orthotospovirus*	*Tomato spotted wilt virus* (TSWV)	Thrips	Midgut	Kritzman et al., 2002
*Fijivirus*	*Southern rice black-streaked dwarf virus* (SRBSDV)	Planthopper	Midgut	Jia et al., 2012b
*Oryzavirus*	*Rice ragged stunt virus* (RRSV)	Planthopper	Midgut	Jia et al., 2012c
*Phytoreovirus*	*Rice dwarf virus* (RDV)	Leafhopper	Filter chamber	Chen et al., 2011
*Phytoreovirus*	*Rice gall dwarf virus* (RGDV)	Leafhopper	Filter chamber	Zheng et al., 2015

The microvilli and basal lamina of insect midgut is the initial barriers for viral entry and dissemination. Thus, many studies have documented that the insect midgut epithelium is a major determinant of vector competence for a variety of persistent-propagative viruses (Hogenhout et al., 2008; Whitfield et al., 2015; Neelakanta and Sultana, 2016; Wei and Li, 2016). The inability of BYSMV to infect the midgut epithelium might due to lack of viral receptor in the midgut, or the innate immune responses in the midgut epithelium that control viral accumulation and block viral transmission (de Assis et al., 2005; Ding, 2010; Donald et al., 2012; Jia et al., 2012a; Chen et al., 2015; Neelakanta and Sultana, 2016; Whitt et al., 2016). Nonetheless, the efficient infection of BYSMV entry and dissemination from the hindgut epithelium allow the *L. striatellus* to be a competent vector for BYSMV transmission.

The hindguts of aphids are initially infected by the majority of species of luteoviruses, which are transmitted by aphids in a non-propagative, circulative manner (Gildow, 1993; Garret et al., 1996; Reinbold et al., 2003; Gray et al., 2014). Unlike the luteoviruses, BYSMV is able to initially infect and efficiently propagate in the epithelial cells of the hindgut and then disseminate into the midgut muscles and other organs (**Figure 3**). Despite the luteoviruses unable to replicate in the vector, long feeding periods on the plants with high virus acquisition facilitate the enough amounts of viruses accumulation and transmission efficiency by the aphids (Gray et al., 1991). In addition, the non-enveloped icosahedral particles of luteoviruses can interact with specific molecules on the surface of the epithelial cells of guts and are likely to enter the epithelial cells by endocytosis (Gildow, 1993). These resulted suggested that different strategies may be employed by the viruses for entry and spread in vectors. Further experiments are needed to determine whether the pattern of the enveloped virions of BYSMV enter the epithelial cells of hindgut via a receptor-mediated endocytic fashion.

## Author Contributions

X-BW and QC conceived and designed the experiments. QC, W-YX, QG, Z-HJ, S-YL, X-DF, and D-MG performed the experiments. X-BW and QC analyzed the data and drafted the manuscript. X-BW and YW participated in experimental coordination and revision of the manuscript. X-BW and QC proofread and finalized the manuscript.

## Conflict of Interest Statement

The authors declare that the research was conducted in the absence of any commercial or financial relationships that could be construed as a potential conflict of interest. The reviewer KJ and handling Editor declared their shared affiliation.

## Supplementary Material

FIGURE S1 Immunofluorescence confocal laser scanning microscopy (iCLSM) signals of BYSMV-infected or healthy SBPHs. Alimentary canals of infected or healthy SBPHs were immunolabeled for BYSMV with N-FITC (green) and stained for actin with phalloidin-rhodamine (red), then examined by confocal microscopy. **(A)** Alimentary canals of BYSMV-infected SBPHs were dissected, isolated, and processed for iCLSM at 12 days padp. **(B)** Alimentary canals of SBPHs exposed to healthy plants were dissected, isolated, and processed for iCLSM at 3, 5, 7, and 12 days padp. Bars, 150 μm.

FIGURE S2 Immunofluorescence confocal laser scanning microscopy signals of BYSMV-infected or healthy SBPHs. The individual fluorescence panels of 1, 2, 3, and 5 padp of **Figure 1** were shown. Bars, 150 μm.

FIGURE S3 Immunofluorescence confocal laser scanning microscopy signals of BYSMV-infected or healthy SBPHs. The individual fluorescence panels of 7, 11, and 12 padp of **Figure 1** were shown. Bars, 150 μm. The bars in hemocytes are equal to 10 μm.

FIGURE S4 Three-Dimensional rendered confocal images. Hindguts of BYSMV-infected SBPHs were processed for iCLSM at 3 **(A)** and 5 **(B)** days padp. Slides of immunolabeled *L. striatellus* hindguts were examined by iCLSM (Olympus FV1000). To study the hindguts and acquire images, a 60X oil-immersion objective was used with detailed scan zoom. At 3 days padp, z-stacks were taken at the three channels with an automatic calculated optimum of 1.09 μm per slide, and 59 slides in total. At 5 days padp, a projection view where 58 optical sections of *L. striatellus* hindgut were merged (*z*-step = 0.85 μm; **B**), a 3-D relief of the hindgut was observable, and structural details of the hindgut were easily discerned. Three-dimensional rendered confocal images were reconstructed with Imaris 7.4.2 software (Bitplane). Bars, 20 μm.

FIGURE S5 The propagation of BYSMV in *L. striatellus*. **(A)** To rule out DNA contamination, the DNase I treated RNA samples without reverse transcript (Non-RT) were used as templates for PCR. The RT products was positive control. All the RNA samples from three independent experiments were examined. **(B)** The accumulations of the mRNAs of BYSMV N, P, and M genes were examined by semi-quantitative RT-PCR in 25 cycles. Actin was used as an internal control. The relative intensity of each lane was quantified by Quantity One software.
