# Supplementary figures and images for: Transmission Characteristics of Barley Yellow Striate Mosaic Virus in Its Planthopper Vector Laodelphax striatellus
Source: Front Microbiol. 2018 Jun 29;9:1419. doi: 10.3389/fmicb.2018.01419 (PMC6034074; doi:10.3389/fmicb.2018.01419)

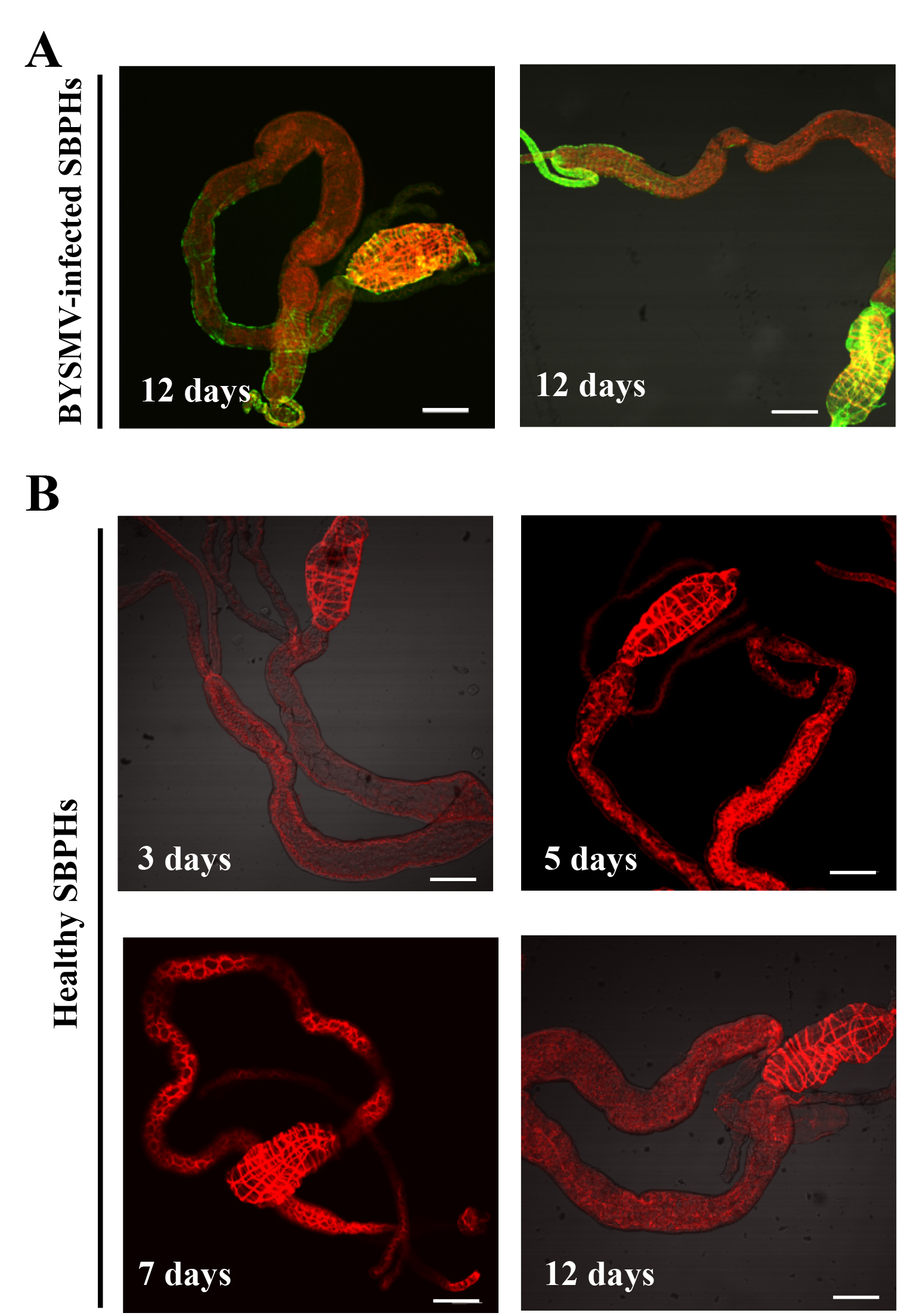

Supplement: FIGURE S1 — Immunofluorescence confocal laser scanning microscopy (iCLSM) signals of BYSMV-infected or healthy SBPHs. Alimentary canals of infected or healthy SBPHs were immunolabeled for BYSMV with N-FITC (green) and stained for actin with phalloidin-rhodamine (red), then examined by confocal microscopy. (A) Alimentary canals of BYSMV-infected SBPHs were dissected, isolated, and processed for iCLSM at 12 days padp. (B) Alimentary canals of SBPHs exposed to healthy plants were dissected, isolated, and processed for iCLSM at 3, 5, 7, and 12 days padp. Bars, 150 μm. [file Image_1.JPEG]

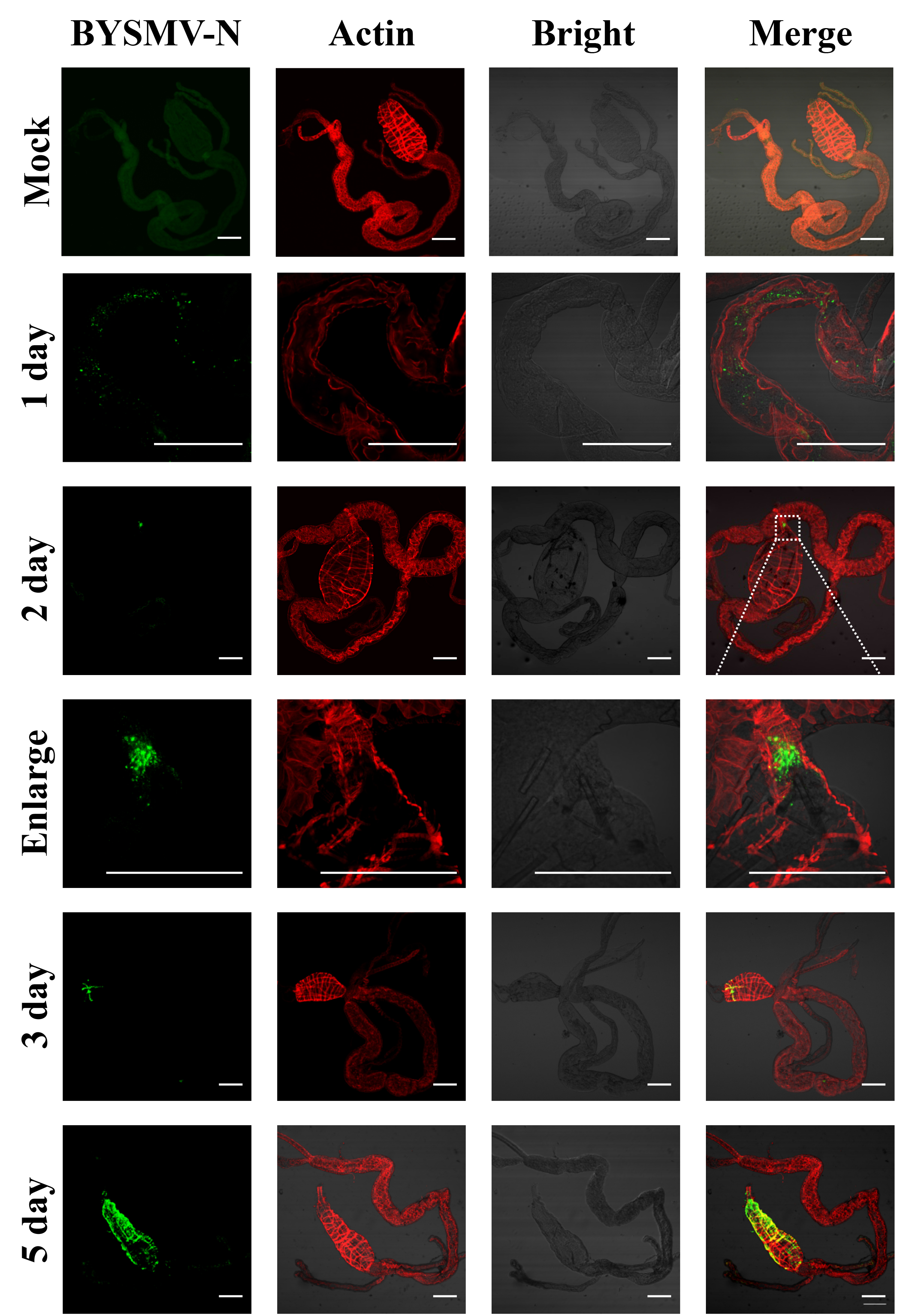

Supplement: FIGURE S2 — Immunofluorescence confocal laser scanning microscopy signals of BYSMV-infected or healthy SBPHs. The individual fluorescence panels of 1, 2, 3, and 5 padp of Figure 1 were shown. Bars, 150 μm. [file Image_2.JPEG]

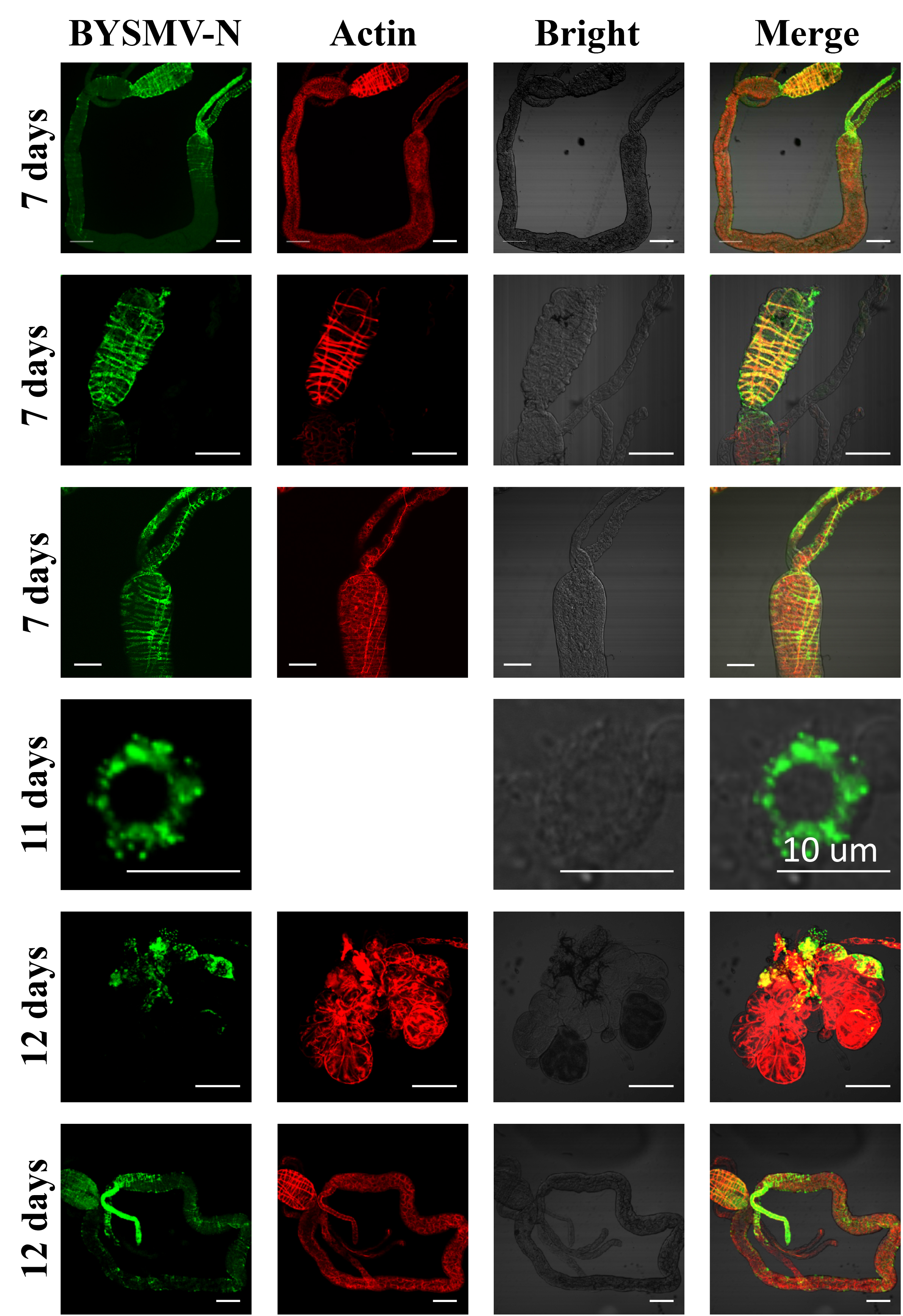

Supplement: FIGURE S3 — Immunofluorescence confocal laser scanning microscopy signals of BYSMV-infected or healthy SBPHs. The individual fluorescence panels of 7, 11, and 12 padp of Figure 1 were shown. Bars, 150 μm. The bars in hemocytes are equal to 10 μm. [file Image_3.JPEG]

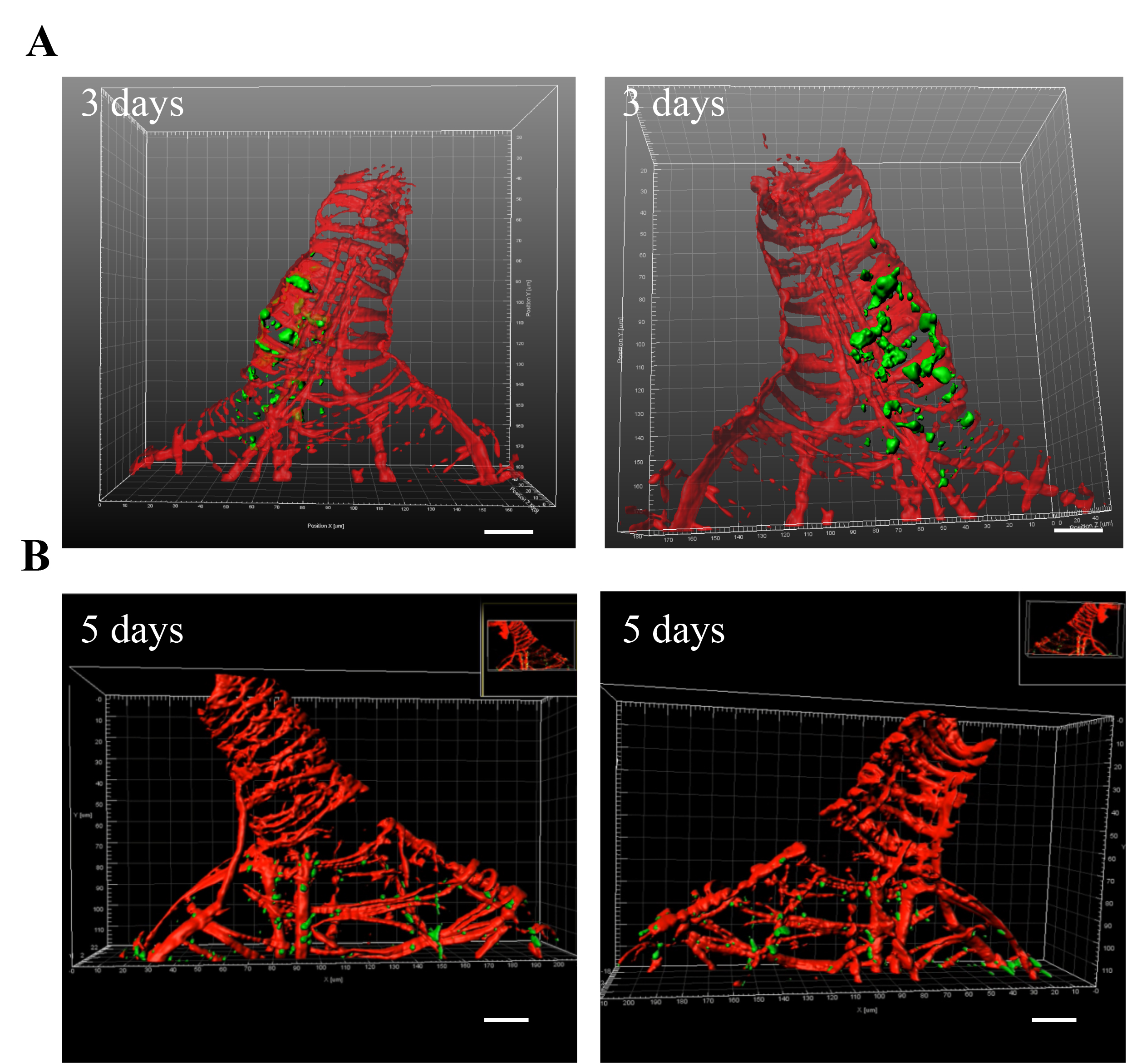

Supplement: FIGURE S4 — Three-Dimensional rendered confocal images. Hindguts of BYSMV-infected SBPHs were processed for iCLSM at 3 (A) and 5 (B) days padp. Slides of immunolabeled L. striatellus hindguts were examined by iCLSM (Olympus FV1000). To study the hindguts and acquire images, a 60X oil-immersion objective was used with detailed scan zoom. At 3 days padp, z-stacks were taken at the three channels with an automatic calculated optimum of 1.09 μm per slide, and 59 slides in total. At 5 days padp, a projection view where 58 optical sections of L. striatellus hindgut were merged (z-step = 0.85 μm; B), a 3-D relief of the hindgut was observable, and structural details of the hindgut were easily discerned. Three-dimensional rendered confocal images were reconstructed with Imaris 7.4.2 software (Bitplane). Bars, 20 μm. [file Image_4.JPEG]

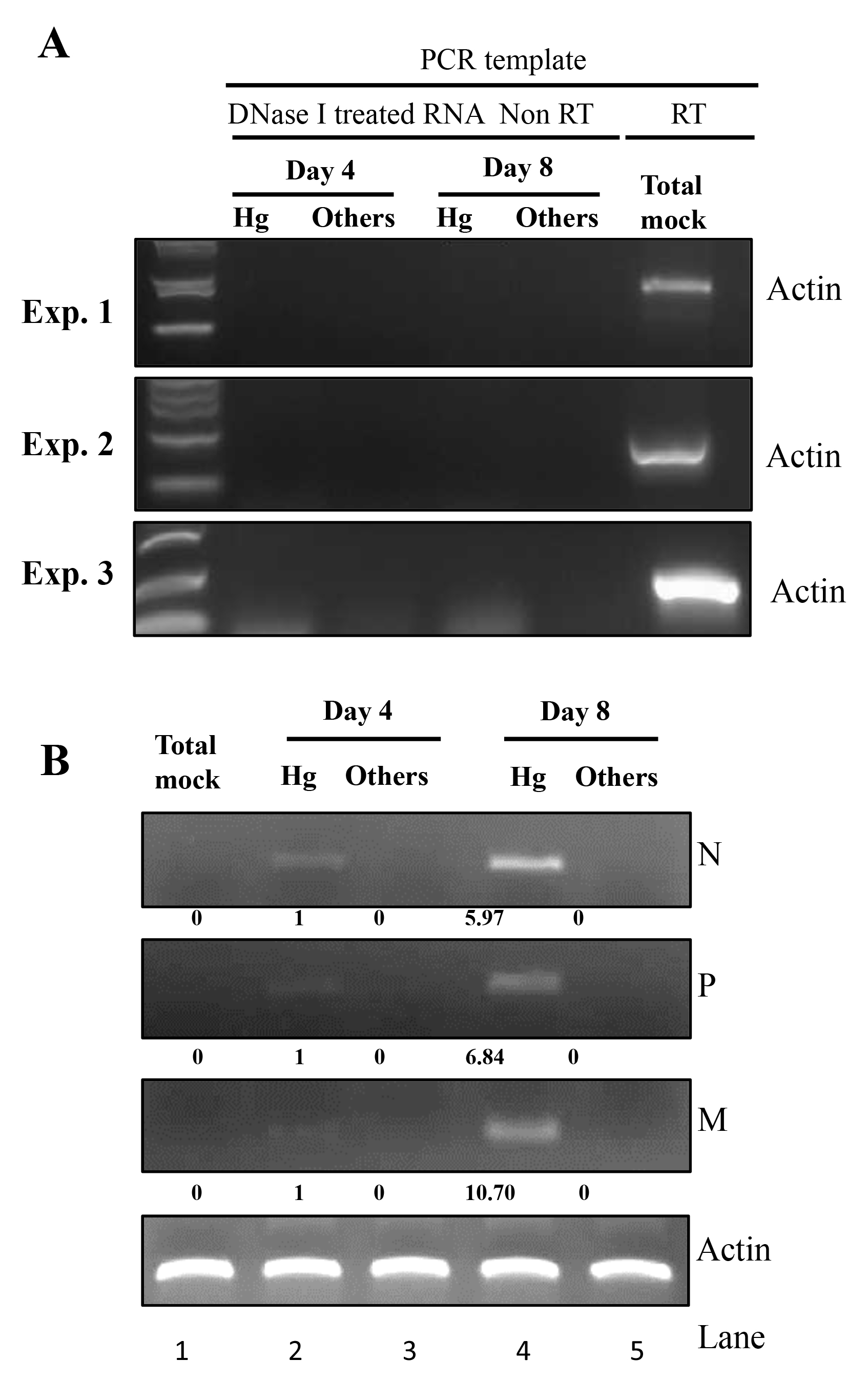

Supplement: FIGURE S5 — The propagation of BYSMV in L. striatellus. (A) To rule out DNA contamination, the DNase I treated RNA samples without reverse transcript (Non-RT) were used as templates for PCR. The RT products was positive control. All the RNA samples from three independent experiments were examined. (B) The accumulations of the mRNAs of BYSMV N, P, and M genes were examined by semi-quantitative RT-PCR in 25 cycles. Actin was used as an internal control. The relative intensity of each lane was quantified by Quantity One software. [file Image_5.JPEG]
